# Multiple Stressors in Vegetable Production: Insights for Trait-Based Crop Improvement in Cucurbits

**DOI:** 10.3389/fpls.2022.861637

**Published:** 2022-05-03

**Authors:** M. S. Parvathi, P. Deepthy Antony, M. Sangeeta Kutty

**Affiliations:** ^1^Department of Plant Physiology, College of Agriculture Vellanikkara, Kerala Agricultural University, Thrissur, India; ^2^Centre for Intellectual Property Rights, Technology Management and Trade, College of Agriculture Vellanikkara, Kerala Agricultural University, Thrissur, India; ^3^Department of Vegetable Science, College of Agriculture Vellanikkara, Kerala Agricultural University, Thrissur, India

**Keywords:** cucurbits, stress tolerance, biotic stress, abiotic stress, metabolic pathways breeding, grafting, mitigation

## Abstract

Vegetable production is a key determinant of contribution from the agricultural sector toward national Gross Domestic Product in a country like India, the second largest producer of fresh vegetables in the world. This calls for a careful scrutiny of the threats to vegetable farming in the event of climate extremes, environmental degradation and incidence of plant pests/diseases. Cucurbits are a vast group of vegetables grown almost throughout the world, which contribute to the daily diet on a global scale. Increasing food supply to cater to the ever-increasing world population, calls for intensive, off-season and year-round cultivation of cucurbits. Current situation predisposes these crops to a multitude of stressors, often simultaneously, under field conditions. This scenario warrants a systematic understanding of the different stress specific traits/mechanisms/pathways and their crosstalk that have been examined in cucurbits and identification of gaps and formulation of perspectives on prospective research directions. The careful dissection of plant responses under specific production environments will help in trait identification for genotype selection, germplasm screens to identify superior donors or for direct genetic manipulation by modern tools for crop improvement. Cucurbits exhibit a wide range of acclimatory responses to both biotic and abiotic stresses, among which a few like morphological characters like waxiness of cuticle; primary and secondary metabolic adjustments; membrane thermostability, osmoregulation and, protein and reactive oxygen species homeostasis and turnover contributing to cellular tolerance, appear to be common and involved in cross talk under combinatorial stress exposures. This is assumed to have profound influence in triggering system level acclimation responses that safeguard growth and metabolism. The possible strategies attempted such as grafting initiatives, molecular breeding, novel genetic manipulation avenues like gene editing and ameliorative stress mitigation approaches, have paved way to unravel the prospects for combined stress tolerance. The advent of next generation sequencing technologies and big data management of the omics output generated have added to the mettle of such emanated concepts and ideas. In this review, we attempt to compile the progress made in deciphering the biotic and abiotic stress responses of cucurbits and their associated traits, both individually and in combination.

## Vegetable Production: The Cucurbit Context

The vegetable crops belonging to the family Cucurbitaceae are known as cucurbits or gourds. This important family of vegetables contains 950 species in over 90 genera and is mainly distributed in the tropics and subtropics (Schaefer and Renner, 2011). Cucurbit family includes several genera and represents the largest tropical vegetable group (Roy and Chakrabarti, 2003; Ebert et al., 2021), as summarized in Table 1

**TABLE 1:** Diverse genera of family Cucurbitaceae.

Genera	Common name	Scientific name
Cucumis	Cucumber	*Cucumis sativus L.*
	Muskmelon or Cantaloupe	*Cucumis melo* L.
Citrullus	Watermelon	*Citrullus lanatus* subsp. *vulgaris* (Schrad.) Fursa
Cucurbita	Winter squash	*Cucurbita maxima* Duchesne
	Summer squash	*Cucurbita pepo* L.
	Pumpkin	*Cucurbita moschata* Duchesne
Benincasa	Wax or Ash gourd	*Benincasa hispida* (Thunb.) Cogn.
Lagenaria	Bottle gourd	*Lagenaria siceraria* (Molina) Standl.
Luffa	Ridge gourd	*Luffa acutangula* (L.) Roxb.
	Sponge gourd	*Luffa aegyptiaca* Mill.
Momordica	Bitter gourd	*Momordica charantia* L.
	Spiny gourd	*Momordica dioica* Roxb. ex Willd
Coccinia	Ivy gourd	*Coccinia grandis* (L.) Voigt
Sechium	Cho cho or Chayote	*Sicyos edulis* Jacq.
Trichosanthes	Snake gourd	*Trichosanthes cucumerina* L.
	Pointed gourd	*Trichosanthes dioica* Roxb.

Wide variability is observed in the genetic makeup of the members with monoploid chromosome number ranging from seven (*Cucumis sativus*) to twenty (*Cucurbita* spp.) (Rai et al., 2007; Choudhary et al., 2016; Samadia and Haldhar, 2017). A great variability also exists in the utilization of these crops, *viz.*, salads (cucumber, gherkins, long melon), sweet dishes (ash gourd, pointed gourd), pickles (gherkins), and desserts (melons). Cucurbit seeds are also high in oil and protein content attesting to their nutritive value (Rahman et al., 2008; Choudhary et al., 2015; Bhargava et al., 2016).

Despite their wide adaptability and varied uses in different parts of the world, their commercial cultivation is increasingly facing the threats of climate change and consequent biotic and abiotic stresses as well as genetic erosion. It is imperative that a holistic approach on the scientific management of various factors affecting the crop performance including development of stress tolerant types, manipulation of metabolic pathways for tolerance/resistance and their crosstalks, as well as propagation strategies for withstanding biotic and abiotic stresses be adopted for successful cucurbit production.

## Biotic Stress Responses in Cucurbits

Cucurbits are often attacked by a wide range of pests including beetles, fruit flies, aphids, white flies, borers, mites etc. (Sharma et al., 2016; Srivastava and Joshih, 2021; Table 2). The pests include those affecting cucurbits worldwide as well as those which are more pronounced in certain regions of the globe, where they attain the status of primary pests. Cucumber moth, *Diaphania indica*, is a potentially damaging pest of cucurbitaceous vegetables worldwide (Chintha et al., 2002; Kinjo and Arakaki, 2002; Hosseinzade et al., 2014; Dai et al., 2018; Jalali et al., 2019; Capinera, 2020; Debnath et al., 2020; Khanzada et al., 2021). The attractiveness of cucurbitaceous host plants for *D. indica* was observed to depend on the species and condition of the plant (uninfested and infested), and sex, mating status and experience of the insect. Females that had experience of cucumber, squash and melon plants were significantly attracted to the same plant, and the larvae were attracted only to volatiles of uninfested cucumber, squash and melon (Jalali et al., 2019). Striped cucumber beetle (StCB) and the western striped cucumber beetle (WStCB) are native to North America and StCB is reported to have attained the status of primary pest in northeastern and midwestern United States and eastern Canada (Haber et al., 2021).

**TABLE 2:** Major pests of cucurbits.

Pest	Scientific name	References
Fruit fly	*Bactrocera cucurbitae*	Haldhar et al., 2014, 2017, 2018
Leaf eating caterpillar	*Diaphania indica*	
Leaf miner	*Liriomyza trifolii*	
Aphids	*Aphis gossypii*	
Ash weevil	*Myllocerus subfasciatus*	
White flies	*Bemisia tabaci*	Wisler et al., 1998; Wintermantel et al., 2019
Beet armyworm	*Spodoptera exigua*	Haldhar, 2016
Red spotted mite	*Tetranychus urticae* (Koch)	Singh and Raghuraman, 2011
Flower beetles	*Mylabris macilenta, Anthicus crinitus*, and *Anthrenus subclaviger*	Haldhar, 2013
Hadda beetle	*Epilachna vigintioctopunctata*	Haldhar et al., 2014, 2017, 2018
Spotted cucumber beetle	*Diabrotica undecimpunctata*	Sharma et al., 2017
Striped cucumber beetle	*Acalymma vittatum*	Sharma et al., 2017
Melon aphid	*Aphis gossypii*	Ng and Perry, 2004
Red pumpkin beetle	*Aulacophora foveicollis (Lucas)*	Khan et al., 2012

Although pests attack affects all stages of cucurbits, severity and susceptibility depends on the plant type and stage of incidence. Pests like red pumpkin beetle and leaf miner are serious at seedling stage (Bains and Prakash, 1985) while beetles [flea beetles (*Phyllotreta cruciferae*) and spotted cucumber beetles (*Diabrotica undecimpunctata*)] were identified as major pests of cucumber at vegetative stage (Alao et al., 2017). Plant growth promoting rhizobacteria (PGPR) induced resistance was reported to be more effective than insecticides for control of cucumber beetles on cucumber possibly by inducing altered production of allelochemicals acting as beetle attractants, repellents, or feeding stimulants (Zehnder et al., 1997). The fruit fly (*Zeugodacus cucurbitae*), a pest of summer squash, cucumber, pumpkin and bitter gourd (Subedi et al., 2021) attacks only flowers and fruits at crop maturity (Ram et al., 2009). Alao et al. (2017) also demonstrated that at vegetative stage of the plant, insect attack was considerably lower in cucumber compared to watermelon, and was attributed to the presence of antixenosis or antibiosis factors in cucumber. Some of the pests that attack cucurbits like whiteflies, thrips and mites, transmit viruses apart from causing feeding damage (Wisler et al., 1998; Park and Lee, 2005; Messelink et al., 2008; Turechek et al., 2014). Although cultivation under greenhouse conditions is reported to be favorable for cucumber production, it is conducive for the rapid development of insect and mite populations (Messelink et al., 2020).

Climate change has led to resurgence of pests and their spread to new areas and often the resistance of varieties breakdown with the evolution of the pest. The fact that pests often become resistant to commercial pesticide formulations in use necessitates a study on pests and their management as well as identification of resistant genotypes and the traits that confer the resistance response.

Cucurbits are found to be affected severely by several diseases including fungal, bacterial and viral diseases, and nematodes, among which the viruses were reported to cause the largest number of diseases (McCreight, 2011; Table 3). Oomycete pathogens like *Pseudoperonospora cubensis*, causing downy mildew, affects all major cucurbit crops, including cucumber, muskmelon, squashes, and watermelon and can assume epidemic proportions (Holmes et al., 2015). Virus diseases, apart from causing reduction in vegetative growth and crop yield, also results in poor fruit quality and makes the plant susceptible to other pathogens as well (Sastry, 2013). However, some studies have demonstrated that healthy wild gourd plants (*Cucurbita pepo* ssp. *texana*) contract bacterial wilt at significantly higher rates than virus infected plants. Prior infection by Zucchini yellow mosaic virus (ZYMV) was found to delay the subsequent onset and progression of bacterial wilt disease by *Erwinia tracheiphila* (Shapiro et al., 2013). Majority of the fungal diseases in cucurbits caused by *Stagonosporopsis cucurbitacearum* (gummy stem blight and black rot), *Alternaria alternata* (leaf spot), *Fusarium solani* (damping off and wilt), *Alternaria cucumerina* (leaf spot) and *Myrothecium roridum* (foliar and stem lesion) are seed borne (Gannibal, 2011; Farrag and Moharam, 2012; Fish et al., 2012). Bacterial pathogens like *Acidovorax avenae* subsp. *citrulli* which causes fruit blotch of cucurbits is also reported to be seed borne and contaminated seeds is the main source of the bacterial inoculum (Block and Shepherd, 2008). Cucurbits adopt various strategies to resist or tolerate diseases. Despite the fact that several genotypes showing resistance to fungal, bacterial, viral and oomycete diseases are available in the germplasm, long-term planting, variable adaptability of pathogens and suppression of host resistance mechanisms by the pathogens often leads to a gradual decline in plant resistance (Chen et al., 2021; Gao et al., 2021; Zhang S. et al., 2021).

**TABLE 3:** Major diseases of cucurbits.

Disease	Causal organism	References
**Fungal diseases**		
Cucurbit powdery mildew	*Podosphaera xanthii*, *Erysiphe cichoracearum*, and *Sphaerotheca fuliginea*	Lebeda et al., 2016
Downy mildew	*Pseudoperonospora* spp.	Lebeda and Cohen, 2011
Anthracnose	*Colletotrichum orbiculare*	Wehner and Amand, 1995
Fruit rot	*Alternaria alternata*, *Fusarium equiseti*, *Fusarium solani*, *Aspergillus* spp., *Phytophthora capsici*, *Penicillium oxalicum*, *Bipolaris* spp., *Botrytis cinerea*, *Cladosporium tenuissimum*	Al-Sadi et al., 2011.
Damping off	*Pythium aphanidermatum, Phytophthora melonis* (in cucumber)	Al-Sadi et al., 2008
Target leaf spot	*Corynespora cassiicola*	Li et al., 2012
*Fusarium wilt*	*Fusarium* spp.	Li et al., 2009
**Bacterial diseases**		
Angular leaf spot	*Pseudomonas syringae*	Bhat et al., 2010
Bacterial wilt	*Erwinia tracheiphila*	Shapiro et al., 2014
Bacterial Fruit Blotch	*Acidovorax citrulli*	Wu et al., 2019
**Viral diseases**		
	Cucumber mosaic virus (CMV)	
	Cucurbit chlorotic yellows virus (CCYV)	Sydänmetsä and Mbanzibwa, 2016
	Squash vein yellowing virus (SqVYV)	Adkins et al., 2008
	Zucchini yellow mosaic virus (ZYMV)	Tsai et al., 2010; Lecoq and Desbiez, 2012
	Watermelon mosaic virus (WMV)	Ayo-John et al., 2014
	Moroccan watermelon mosaic virus (MWMV)	Arocha et al., 2008
	Papaya ringspot virus (PRSV)	Omar et al., 2011
	Cucumber green mottle mosaic virus (CGMMV)	Molad et al., 2021
**Parasites**		
*Root Knot disease*	*Meloidogyne* spp.	Omar and Adam, 2018

## Abiotic Stress Responses in Cucurbits

Cucurbits are a vast group of vegetables which contribute to the daily diet of a large portion of the world population and are grown almost throughout the world. These vegetables are a good source of nutrients and hence play a vital role in ensuring nutritional security to mankind. Increasing food demands call for intensive, offseason and year-round cultivation of cucurbits, thereby predisposing these crops to a multitude of stressors like high temperature, drought, salinity, heavy metal toxicity, nutrient deficiency/toxicity, soil pH etc. The climate change scenario has further intensified the predisposition to abiotic stressors- high temperature, drought and salinity being the major players in the global arena.

Plants support their growth and development even under adverse conditions by developing several tolerance and adaptation mechanisms. The biochemical, physiological and molecular responses elicited in response to abiotic stress are guided by common stress tolerance pathways, shared by most of the cultivated crops.

### Heat Stress Response

Global warming is one of the most alarming effects of climate change with a long term impact on agriculture, particularly the vulnerable vegetable crops. Heat stress is a function of temperature, duration/period of stress and rate of increase in temperature. Cucurbits being warm season vegetables, are more likely to be exposed to heat stress particularly the summer crop. High temperatures can influence cell development, synthesis of cell wall, plant hormonal connections, amalgamation of proteins, stomatal regulation (thereby influencing photosynthesis, CO_2_ assimilation and respiration) etc. (Hasanuzzaman et al., 2012).

High temperatures adversely affect several physiological, biochemical, morphological and molecular processes and pathways in plants. Seed germination of cucumber and melon is reduced drastically at 45 and 42°C respectively (Kurtar, 2010). The ideal temperatures for crop growth and development are 18.3–23.8°C for squash, pumpkin, muskmelon and cucumber, and 23.8–29.4°C for watermelon. In cucumber (*Cucumis sativu*s L.) or watermelon (*Citrullus lanatus* L.), temperatures above 35°C caused a reduction in flowers and sugar content (Lai et al., 2018). Heat stress resulted in reduced biomass, root growth and development, leaf area (Porter and Gawith, 1999; Al-Busaidi et al., 2012; Balal et al., 2016) and decreased fruit length, fruit diameter and reduced fruit weight (Balal et al., 2016). The alterations in cell division, cell elongation, water loss and reduced photosynthetic rate under heat stress resulted in reduced yield, leaf area, biomass etc. (Hasanuzzaman et al., 2013).

The photosynthetic rate is positively correlated with the chlorophyll content in the leaves (Lin et al., 2011). Plant growth and yield are adversely affected due to reduced chlorophyll under high temperature stress and subsequent reduction in photosynthetic rate. In cucumber, heat stress induced reduction in chlorophyll and photosynthetic rate has been observed (Balal et al., 2016; Zhou et al., 2016). Reactive oxygen species (ROS) levels are enhanced in the plant tissues in response to heat stress which results in oxidative stress (Suzuki and Mittler, 2006; Potters et al., 2007; Pucciariello et al., 2012). During the electron transport in photosynthetic process, electron leakage to oxygen molecule results in generation of ROS (Sharma et al., 2012). Plants have different mechanisms (enzymatic and non-enzymatic) to detoxify the ROS. Several antioxidative enzyme activities, i.e., superoxide dismutase (SOD), peroxidase (POD), catalase (CAT), ascorbate peroxidase (APX), guaiacol peroxidase (GPX), glutathione reductase (GTR), monodehydroascorbate reductase (MDHAR), etc. are generally reduced under heat stress (Balal et al., 2016) and are upregulated in response to stress particularly in tolerant species or in response to ameliorants (Balal et al., 2016).

Another strategy to counteract stress induced osmotic damage is the accumulation of various compatible solutes like proline, glycine betaine (GB), amino acids, sugars, quaternary ammonium and sulphonium compounds etc. (Majumder et al., 2009). In cucumber, increased levels of proline, glycine betaine and total soluble sugars were reported in response to heat stress (Balal et al., 2016).

Screening of cucurbit genotypes based on these traits is an effective strategy to identify stress tolerant lines/varieties. High temperature tolerant varieties have been developed in cucurbits such as AHW-19, AHW-65, Thar Manak (Watermelon), Thar Samridhi (bottle gourd), Thar Karni (ridge gourd), Thar Tapish (sponge gourd) etc. (Saroj and Choudhary, 2020).

### Drought Stress Response

Cucurbits are warm season vegetable crops mostly cultivated in the summer season, hence prone to drought stress if not irrigated at critical stages of growth. Drought response is classified into three categories *viz.*, drought escape (shortening the life cycle), drought avoidance (minimizing water loss or maximizing water uptake thereby preventing exposure to stress) and drought tolerance (helps the plant to withstand stress by osmoregulation, osmotic adjustment, stomatal regulation etc.). However, crop adaptation to drought may be achieved through a balance between these three strategies (Saroj and Choudhary, 2020). Hence, a combination of different traits should be used as a screening criterion for drought tolerance, rather than a single trait (Singh and Sarkar, 1991). The important traits to be considered while breeding for drought tolerance are early vigor, root depth and density, low and high temperature tolerance, carbon isotope discrimination, osmoregulation, low stomatal conductance, leaf posture, reflectance and duration, sugar accumulation etc. However, priority should be given to those traits which can maintain stability of yield in addition to overall yield (Parry et al., 2005). Some of the drought tolerant genotypes identified are AHW-65 and Thar Manak in watermelon, VRSM-58, AHS-10, AHS-82 in snapmelon etc. In cucumber, drought stress reduces photosynthetic rate, increases superoxide anion radicals (O_2_^.–^), electrolyte leakage and lipid peroxidation products like malondialdehyde (MDA), whereas the activities of key antioxidant enzymes superoxide dismutase (SOD) and peroxidase (POD) as well as soluble sugar and proline contents are decreased (Wang J. et al., 2012; Zhang et al., 2013; Fan et al., 2014; Sun et al., 2016).

### Salinity Stress Response

Soil salinity has become a severe problem in agricultural production. It is one of the major factors limiting plant growth and productivity particularly in the arid and semi-arid regions of the world (Parida and Das, 2005). Under salinity conditions, stress is induced due to lower water potential of the root medium, toxic effects of Na^+^ and Cl^−^ and nutrient imbalance by reduction in uptake or shoot transport (Colla et al., 2006). Salinity stress response is multigenic, as a number of processes involved in the tolerance mechanism are affected, such as various compatible solutes/osmolytes, polyamines, ROS and antioxidant defense mechanisms, ion transport and compartmentalization of injurious ions (Sairam and Tyagi, 2004).

In cucumber, the salt tolerance in a genotype was associated with higher relative water content (RWC), total chlorophyll content, and SOD, CAT, and APOX activities, together with the lower MDA and proline contents, and Na^+^ and Cl^–^ concentrations (Furtana and Tipirdamaz, 2010). Sodium chloride stress induces reduction in biomass, photosynthetic pigments, and proline accumulation, while lipid peroxidation and K^+^, Na^+^, and Cl^–^ contents are increased (Hawrylak-Nowak, 2009). The addition of 150 mM of NaCl to the nutrient solution of a floating system where 30 varieties of Cucurbitaceae species were cultivated, affected plant growth parameters (number of leaves, shoot length, diameter and dry weight, root length and dry weight) in a genotype-dependent manner (Modarelli et al., 2020). Salinity reduced chlorophylla content by up to 49% in some genotypes, whereas in others chlorophylla content increased by up to 61%. Similarly, chlorophyllb was reduced by salinity by up to 51% in some genotypes or increased by up to 64% in some others. The increase in photosynthetic pigments was considered as a consequence of the reduction of the leaf area and therefore of the dilution effect. Moreover, salinity increased electrolyte leakage by up to 509%, as compared to the non-salinized control.

### Heavy Metal Toxicity

Heavy metal accumulation in soils is of great concern in agricultural production due to the adverse effects on food safety and marketability, crop growth due to phytotoxicity, and environmental health of soil organisms (Gill, 2014). Heavy metals cause irreversible damage to a number of vital metabolic constituents and important biomolecules including injury to plant cell walls and cell membranes. Mercury, lead, cadmium, vanadium, arsenic, chromium etc. are some of the heavy metals which are present as soil pollutants and cause severe damage to the crops raised. A common consequence of heavy metal toxicity is the excessive accumulation of ROS and methylglyoxal (MG), both of which can cause peroxidation of lipids, oxidation of protein, inactivation of enzymes, DNA damage and/or interact with other vital constituents of plant cells.

Mercury (Hg) and lead (Pb) heavy metal stress results in high peroxidase activity in cucumber, bottle gourd, sponge gourd and bitter gourd (Khan and Chaudhry, 2006, 2010). In melon, with increasing cadmium concentration, seedling growth, net photosynthetic rate (P_n_), stomatal conductance (g_s_), transpiration rate (T_r_), and stomatal limitation (L_s_) decreased; meanwhile, intercellular carbon dioxide concentration (C_i_) increased significantly (Zhang et al., 2016; Khan et al., 2020). Hg induces oxidative stress in cucumber seedlings, resulting in plant injury due to reduced activities of antioxidant enzymes (catalase and ascorbate peroxidase), reduced chlorophyll content, increased lipid peroxidation, protein oxidation etc. (Cargnelutti et al., 2006).

## Trait Gene Discovery and Functional Annotation- Individual and Combined Stress Events

Trait based crop improvement assumes significance in the context of the highly variable environmental conditions, which often results in the co-existence of multiple stresses. Identification of suitable traits/trait-combinations conducive for conferring tolerance in different ecosystems is inevitable. There have been promising reports on the functional characterization of different stress responsive genes in cucurbits or the genes cloned from cucurbits in model crops (Parmar et al., 2017; Nanasato and Tabei, 2020). The High Affinity K^+^ Transporter (HKT) genes encode Na^+^ and/or K^+^ transport systems, active at the plasma membrane and play a crucial role in imparting salt tolerance to different plant species e.g., *HKT 1;5* in barley (Hazzouri et al., 2018) and *CmHKT1;1* in pumpkin (Fu et al., 2018). The YUCCA proteins are critical partners in auxin biosynthesis in plants, which have been reported to regulate response to abiotic stresses and flower development in cucumbers. *CsYUC8* and *CsYUC9* were specifically upregulated to elevate the auxin level under high temperature in *Cucumis sativus*. *CsYUC10b* was dramatically increased but *CsYUC4* was repressed in response to low temperature. *CsYUC10a* and *CsYUC11* act against the upregulation of *CsYUC10b* under salinity stress, suggesting that distinct YUC members participate in different stress responses, and may even antagonize each other to maintain the proper auxin levels in cucumber (Yan et al., 2016). A wholistic genomic and functional analysis of *bHLH* genes was attempted in cucumber to identify 142 bHLH genes, classified into 32 subfamilies, among which five *CsbHLH* genes were found to simultaneously respond to three abiotic stresses (NaCl, ABA and low-temperature treatments). Targeted promoter analysis also revealed many *cis*-elements responsive to multiple stresses and plant hormones (Li et al., 2020). Similar attempts targeting different traits have resulted in the identification of prospective candidate genes, which have been functionally characterized in either cucurbits or in model crops like Arabidopsis. There have been reports on novel candidates such as intrinsically disordered proteins belonging to Plant Group II LEA Proteins with possible roles in multiple stress responses (Abdul Aziz et al., 2021), which could be promising even in cucurbits. The functional annotation of candidate genes in cucurbits has been achieved by traditional over-expression or gene silencing approaches, with recent advancements leading to the adoption of advanced gene interference technologies and CRISPR/CAS mediated gene editing approaches. The successful trait-gene based crop improvement attempts in this direction in different cucurbits have been tabulated in Table 4.

**TABLE 4:** Trait-gene discovery and functional or translational characterization in cucurbits.

Gene	Source	Crop	Target Trait	Remarks
*Cbf1*	*Arabidopsis thaliana*	Cucumber (OE)	Chilling tolerance	Marker free Gupta et al., 2012
*CsWAX2*	Cucumber	Cucumber (OE)	Abiotic and biotic stress response	Wang et al., 2015
*CsATAF1*	Cucumber	Cucumber (RNAi)	Drought stress tolerance	Wang J. et al., 2018
*CsCaM3*	Cucumber	Cucumber (OE)	High temperature stress tolerance	Yu et al., 2018
*CsYUC11*	Cucumber	*Arabidopsis thaliana* (OE)	Salinity tolerance	Yan et al., 2016
*CsbHLH041*	Cucumber	Cucumber *and Arabidopsis thaliana* (OE)	Salinity and ABA tolerance	Li et al., 2020
*CMV 2a/2b*	Watermelon	Artificial microRNAs	Virus resistance	Liu et al., 2016
*CsGPA1*	Cucumber	Cucumber (RNAi)	Drought stress tolerance	Liu et al., 2021d
*CmRCC1*	Pumpkin	Tobacco (OE)	Cold stress tolerance	Wang et al., 2021
Chimeric gene construct containing truncated ZYMVcp and PRSV W cp genes	*Citrullus lanatus* Watermelon	*Citrullus lanatus* Water melon (RNAi)	Virus resistance	Yu et al., 2011
*CMV replicase*	Defective viral genome mediated resistance against CMV	Lilium	Virus resistance	Azadi et al., 2011
*eIF4E*	Cucumber	Cas9/subgenomicRNA (sgRNA technology)	Virus resistance	Chandrasekaran et al., 2016
*RBOHD*	Pumpkin	CRISPR/Cas9-mediated mutagenesis	Salinity tolerance	Huang et al., 2019
*RBOHD*	Pumpkin	*Arabidopsis thaliana* (OE)	Salinity tolerance	Huang et al., 2019
*CsWIP1*	Cucumber	CRISPR/Cas9-mediated mutagenesis	Gynoecious phenotype	Hu et al., 2017
*ZW-20*	Squash	Cucumber	*Zucchini yellow mosaic virus resistance*	Fuchs and Gonsalves, 1995
*CZW-3* (CMV, ZYMV, and WMV2)	Squash	Cucumber	Virus resistance	Fuchs and Gonsalves, 1997

*OE, overexpression; RNAi, RNA interference.*

## Possible Crosstalk in Pathways/Mechanisms Under Combined Stresses

Challenges faced by plants come in multitudes and often a combinatorial response to the simultaneous occurrence of stresses, either abiotic or biotic or cross combinations, is actually displayed by plants. A concerted effort to study cucumber plants exposed to salt stress and thereafter infected with *Pseudomonas syringae* pv *lachrymans* (*Psl*), revealed that there were distinct changes in photochemistry, the antioxidant system, primary carbon metabolism, salicylic acid (SA) and abscisic acid (ABA) contents. The careful examination of hormonal and redox balance as well as the carboxylate metabolism and activities of some NADPH-generating enzymes indicated that salt-stressed plants were more prone to pathogen infection. There can be critical convergence points and master regulators for the characteristic response to specific abiotic factor-pathogen combination. In case of cucumber, the combinatorial stress response to salt stress and *P. syringae* is dominated by the abiotic factor. Modulation of SA-mediated defense, hormonal, ROS/redox and metabolic signals are responsible for predisposing cucumber plants to *P. syringae* after sequential salt stress episodes making them highly susceptible (Chojak-Koźniewska et al., 2018). Another important stressor is temperature which has profound influence on the occurrence of bacterial diseases caused by *Ralstonia solanacearum* (causal agent of wilt in tomato), *Acidovorax avenae* (causal agent of seedling blight and bacterial fruit blotch of cucurbits) and *Burkholderia glumae* (causal agent of bacterial panicle blight in rice) (Kudela, 2009; Pandey et al., 2017). *Cucumber mosaic virus* (CMV) as an important viral invader of cucurbits has significance in understanding its interactive specificities with other stressors. CMV infection was found to impart improved drought tolerance of *Capsicum annum* (pepper), *S. lycopersicum*, and *Nicotiana tabacum* (tobacco) (Xu et al., 2008; Pandey et al., 2017). The combinatorial effects between abiotic-abiotic and abiotic-biotic pairs can be starkly different. Generally abiotic stress combinations can have “only net effects and no stress interactions,” leading to additive deteriorative effects due to co-occurrence of two stresses together. It will be different for a plant-pathogen system wherein, it may lead to enhanced or reduced susceptibility to the pathogen; some pathogens also modulate abiotic stress tolerance. In case of heat–pathogen and drought–pathogen stress combinations, wherein multiple individual stresses or sequential stresses occur one after the other, either prior priming leading to stress memory or predisposition can be the consequence (Pandey et al., 2017). *CsbHLH041* is an important regulator in response to multiple abiotic stresses like salinity and water deficit in cucumber (Li et al., 2020). Phytohormonal variations can result in a common response against both biotic and abiotic stressors such as similar morphological root changes under CMV infection and a heavy metal challenge like cadmium stress (Vitti et al., 2013). It was very recently identified that there are distinct metabolite signatures, with special reference to amino acids, associated with response to salt and drought stresses in *Cucumis melo* L. (Chevilly et al., 2021). High histidine contents and the ability to sequester salts in vacuoles are expected to confer salt stress tolerance capacity. However, varieties or cultivars with enhanced levels of isoleucine, glycine, serine and asparagine exhibited drought stress tolerance. There was a retardation in tolerance to abiotic stresses when the phenylalanine levels were high (Chevilly et al., 2021).

## Possible Strategies to Achieve Combined Stress Tolerance

### A. Propagation Methods as a Means for Combining Multiple Stress Tolerance Traits

Vegetable grafting is a unique horticultural technique used in the propagation of fruit vegetables due to the multitude of advantages over the conventional propagation methods. Vegetable grafting was primarily developed and practiced with an objective of avoiding the damage caused by soil borne pathogens and pests (Cohen et al., 2007). The scope for grafting has further widened for combating abiotic stress tolerance, with the advancement in our understanding of the rootstock mediated effect on superior performance of scion, exploiting the physiological stress tolerance reserved in the wild species (Colla et al., 2010, 2012). Grafting has emerged as a viable alternative to relatively slower breeding approaches for enhancing environmental stress tolerance in fruit vegetables (Flores et al., 2010). Grafting is a special method of adapting plants to counteract environmental stresses by grafting superior commercial cultivars onto specific vigorous rootstocks (Lee and Oda, 2003).

Cucurbits are the first group of vegetables where grafting was widely popularized to combat biotic stress particularly Fusarium wilt. Research on cucurbit grafting began in the 1920s with the use of *Cucurbita moschata* as a rootstock for watermelon in Japan. Grafting is a quick, less expensive and viable solution for combating soil borne pathogens and their novel races, in comparison to the tedious breeding approach adopted for developing resistant cultivars (Davis et al., 2008). Watermelon, cucumber and melons are the major cucurbits which are propagated using grafted seedlings in order to overcome biotic and abiotic stresses.

#### (a) Grafting for Biotic Stress Tolerance in Cucurbits

In cucurbits, grafting has proven to impart resistance/tolerance to several fungal, bacterial and nematode infections, soil borne pathogens and even some viral as well as foliar pathogens. The most devastating soil borne pathogen of cucurbits is *Fusarium oxysporum* causing Fusarium wilt (FW). In watermelon and cucumber, grafting is the most popular alternative for controlling Fusarium wilt. Some of the achievements in combating biotic stress in cucurbits through grafting has been summarized in Table 5.

**TABLE 5:** Rootstocks for combating biotic stress in cucurbits.

Sl No.	Crop/Scion	Rootstock	Stress tolerance imparted	Region	Condition	References
1	Watermelon	*Lagenaria siceraria* (16S-71)	*Fusarium oxysporum* f. sp. *niveum*	China	Open field	Zhang M. et al., 2021
2	Oriental melons (cv. makuwa) and pickling melon	Cucurbita moschata (Shirokikuza) and *C. maxima* × *C. moschata* (Shintosa)	*Fusarium oxysporum*	Japan	Open field	Sakata et al., 2008
3	Watermelon	*Citrullus* sp. (RS-18, RS-10, RS-11)	*Fusarium oxysporum*	Bangalore (India)	Open field	Pal et al., 2020
4	Watermelon	*L. siceraria* (WMXP-3938)	*Phytophthora capsici*	United States	Open field	Kousik et al., 2012
5	Watermelon (cv. Fiesta)	*C. lanatus* var. *citroides* (RKVL 315 and 318)	Nematode *(Meloidogyne incognita)*	–	Open field	Thies et al., 2010
6	Cucumber	*Cucurbita maxima* and *C. moschata*	*Fusarium oxysporum; Pythium aphanidermatum*	Egypt	Open field	Reyad et al., 2021
				Oman	Green house	Deadman et al., 2009
7	Cucumber (cv. Caspian 340)	*C. maxima*	*Pythium aphanidermatum*	-	Open field	Rostami et al., 2015
8	Cucumber (cv. Centenario)	*Lagenaria siceraria* (Lag 53)	Nematode *(Meloidogyne incognita)*	Mexico	Green house	Suárez-Hernández et al., 2021
9	Cucumber	*Benincasa hispid*a	Black root rot (*Phomopsis sclerotioides*)	–	–	Yamaguchi and Iwadate, 2009
10	Cucumber, melon and watermelon	*Cucumis pustulatus*	Nematode (*Meloidogyne incognita*) and Fusarium wilt	China	Green house	Liu et al., 2015
11	Melon	*Cucumis melo, Cucurbita maxima* × *Cucurbita moschata*	*Fusarium oxysporum*	South Korea Italy	Open field	Lee, 1994; Nisini et al., 2002
12	Inodorous melon	*Cucurbita maxima* Duchesne × *Cucurbita moschata* Duchesne (RS841, P 360, ES99-13, Elsi)	*Fusarium oxysporum* f. sp. *melonis* and *Didymella bryoniae*	Italy	Green house	Crino et al., 2007
13	Honey dew melon (cv. Honey yellow) Galia melon (cv. Arava)	*Cucumis metulifer* line (USVL-M0046)	Root knot nematode *Meloidogyne* spp.	Florida (United States)	Green house	Guan et al., 2014
14	Oriental Melon	*C. moschata, C. metuliferus*, and *Sicyos angulatus*	Fusarium wilt	–	–	Davis et al., 2008
15	Bitter gourd	*Luffa aegyptiaca*	*Fusarium oxysporum*	–	Open field	Lin et al., 1998
16	Bitter gourd	*Citrullus colocynthis, Cucumis metuliferus, Cucurbita moschata*	Nematode (*Meloidogyne incognita*)	Tamil Nadu (India)	Glass house	Tamilselvi et al., 2017

#### (b) Grafting for Abiotic Stress Tolerance in Cucurbits

Grafting has also emerged as an effective adaptive technique to overcome abiotic stressors including drought, flooding, waterlogging, salinity, heavy metal contamination, suboptimal and supraoptimal temperatures, nutrient deficiencies, toxicities etc. When the plants are exposed to these abiotic factors beyond the threshold level for optimal biochemical/physiological activity or morphological development, it results in reduction of plant performance and subsequent yield reduction. In cucurbits, several studies involving different rootstocks have proven their efficiency in alleviating the adverse effects of a number of abiotic factors; some of these have been summarized in Table 6.

**TABLE 6:** Rootstocks for combating abiotic stress in cucurbits.

Sl No.	Crop/Scion	Rootstock	Stress tolerance imparted	Region	Condition	References
1	Cucumber	*Luffa cylindrica*	Drought	China	Growth chamber	Liu, 2016
2	Cucumber	*Cucurbita moschata*	Salinity; low temperature	China	Open field	Niu et al., 2019
				China	Open field	Zhu et al., 2008
				Japan	Green house	Shibuya et al., 2007
3	Cucumber	Figleaf gourd (*Cucurbita ficifolia* Bouché) and bur cucumber (*Sicyos angulatus* L.)	Low temperature	–	–	Schwarz et al., 2010
	Cultivar ‘Infinity’	Figleaf gourd (*Cucurbita ficifolia*), bottle gourd (*Lagenaria siceraria* cv. Sharda)	Low temperature	Jodhpur (India)	Unheated green house	Kumar et al., 2019
4	Cucumber (cv. Jinyou No. 35)	*Momordica charantia* L. (Changlv)	Heat Stress tolerance	China	Plastic arched shed	Xu et al., 2018
5	Cucumber (cv. Ekron)	*C. sativus* L*., C. maxima x C. moschata*	Salinity tolerance	Italy	Green house	Colla et al., 2013
	(cv. Jinchum No. 2)	*Cucurbita ficifolia*		China	Green house	Huang et al., 2010
6	Cucumber (cv. Gian Co F1)	VSS-61 F1 *Cucurbita pepo* (squash) and Ferro *Cucurbita maxima* × *C. moschata*	Heat and Salinity stress	Egypt	Net house	Bayoumi et al., 2021
7	Cucumber (cv. Jinyou 35)	*Cucurbita moschata* (Jinmama 519)	Chilling tolerance	China	–	Fu et al., 2021
8	Cucumber (cv. Akito)	*C. maxima* x *C. moschata* Shintoza	Copper toxicity	Italy	Green house	Rouphael et al., 2008b
	(cv. Creta)	*C. maxima* × *C. moschata* Power	Ni and Cd toxicity	–	–	Savvas et al., 2013
	(cv. Ekron)	*C. maxima* × *C. moschata* (P360)	Acidity and Al toxicity	Italy	Green house	Rouphael et al., 2016
9	Cucumber (cv. Sharp 1, cv. Natsubayashi)	*Cucurbita sp.* (Shintosa-1gou, Hikaripower-gold, Yuyuikki-black)	Organic pollutant (dieldrin)	Japan	Open field	Otani and Seike, 2007
10	Watermelon (cv. Crimson tide)	*Lagenaria siceraria*	Flooding tolerance	Turkey	Green house	Yetisir et al., 2006
11	Watermelon (cv. Ingrid)	*Cucurbita maxima × Cucurbita moschata* (PS1313)	Drought tolerance	Italy	Green house	Rouphael et al., 2008a
	(cv. Zaojia 8424)	(Qingyan zhenmu No. 1)	Nitrogen use efficiency	–	Open field	Nawaz et al., 2017
	(cv. Crimson Sweet)	*C. maxima* × *C. moschata* (Shintoza) *Citrullus colocynthis* (L.) Schrad (Esfahan)	Drought	Italy	Green house	Bikdeloo et al., 2021
12	Watermelon (cv. Mahbubi)	*Cucurbita pepo* (Tiana F1 hybrid); *Cucurbita maxima*	Cd toxicity	Iran	Green house	Shirani-Bidabadi et al., 2018
13	Watermelon (cv. Zaojia 8424)	*Cucurbita maxima × Cucurbita moschata* (Qingyan zhenmu No. 1) and *Lagenaria siceraria* (Jingxinzhen)	Vanadium toxicity	China	–	Nawaz et al., 2018
14	Bitter gourd	*Cucurbita moschata*	Low temperature	United States	Green house	Wang J. et al., 2018
15	Bitter melon (cv. New Known You #3)	*Luffa cylindrica* (cv. cylinder #2) *Momordica charantia*	Flooding tolerance	China China	Pot study Pot study	Liao and Lin, 1996; Peng et al., 2020

Under the present climate change scenario multiple stresses in combination or separately, pose severe threat to vegetable production including cucurbits. Use of rootstocks conferring multiple stress resistance could be a sustainable and eco-friendly alternative to the more complicated traditional/molecular breeding approaches to develop multiple stress resistant varieties (Table 7). The melon hybrid (*Cucurbita maxima x Cucurbita moschata*), figleaf gourd (*Cucurbita ficifolia*), pumpkin, bottle gourd and sponge gourd rootstocks have the potential to impart multiple stress tolerance to scions of different cucurbits. Other wild and cultivated species of cucurbits could also be explored for their capabilities to confer multiple stress resistance to susceptible species/varieties.

**TABLE 7:** Prospective rootstocks for multiple stresses tolerance interventions.

Sl No.	Rootstock*	Crops	Biotic/abiotic stress tolerance
1	*Cucurbita maxima x Cucurbita moschata*	Cucumber	Fusarium wilt, Pythium, salinity, heat, Ni, Cd, Al toxicity, acidity.
		Watermelon	Fusarium wilt, drought, Nitrogen use efficiency, Vanadium toxicity
		Melon	Fusarium wilt, *Didymella bryoniae*
2	*Cucurbita moschata*	Bitter gourd	Nematode, low temperature
		Cucumber	Salinity, low temperature
		Oriental melon	Fusarium wilt
3	*Lagenaria siceraria*	Watermelon	Fusarium wilt, *Phytophthora capsici*, Flooding, Vanadium toxicity
		Cucumber	Nematode (*Meloidogyne incognita*)
4	*Luffa cylindrica*	Cucumber	Drought
		Bitter gourd	Flooding
5	*Cucurbita ficifolia*	Cucumber	Low temperature, salinity

*The information on the rootstock can be derived from Tables 5, 6.

### B. Genetic Manipulation Avenues for Developing Stress Tolerance (Conventional/Molecular Breeding/Biotechnological)

#### (a) Breeding for Biotic Stress Tolerance

Screening of germplasm for resistance, utilization of the identified resistant lines as donors for recombination breeding or backcross breeding, interspecific crosses, mutation breeding and manipulation using propagation strategies are the widely used conventional strategies for the development of biotic stress tolerant cucurbit genotypes. Cucurbits are widely affected by viral diseases and among them bottle gourd is found to be moderately resistant to viral disease caused by CMV and yellow mosaic virus (ZYMV) (Provvidenti and Gonsalves, 1984; Provvidenti, 1995; Ling and Levi, 2007). It also displays resistance to fungal diseases like Fusarium wilt (Yetişir and Sari, 2003) and powdery mildew (Kousik et al., 2008) and has been exploited in its use as rootstock for watermelon (Yetişir and Sari, 2003; Keinath and Hassell, 2014).

Resistance to viruses as well as other pests and diseases has also been identified in wild or semi-domesticated types of bitter gourd (*M. charantia var. muricata*) (Asna, 2018). Previous studies also demonstrated the utility of mutation breeding in the development of biotic stress tolerant genotypes. Bitter gourd cultivar MDU 1 developed through mutation breeding from the landrace MC 013, displayed tolerance to pumpkin beetle, fruit fly and leaf spot diseases (Rajasekharan and Shanmugavelu, 1984). The reported cucurbit genotypes resistant to specific pathogens/pests are tabulated in Table 8.

**TABLE 8:** Biotic stress resistant genotypes identified cross different cucurbits.

Pathogen/pest	Crop	Resistant genotype identified	References
Tomato leaf curl New Delhi virus	*Luffa cylindrica Roem.*	DSG-6, DSG-7, DSG-9, and DSG-10	Islam et al., 2010
	*L. cylindrica (*L.) *Roem*	IIHR-137, IIHR-138, IIHR-Sel-1	Kaur et al., 2021
Potyviruses	*Cucumis melo*	PI 414723 and PI 124112	Martín-Hernández and Picó, 2021
Mosaic diseases	*M. charantia var. muricata*	IC 213312, AC-16/1, AC-16/4, AC-16/9, and AC-16/21	Asna, 2018
Broad spectrum virus diseases	*Lagenaria siceraria*	USVL#1-8 and USVL#5-5	Ling et al., 2013
Fruit rot	*Cucumis sativu*s L.	PI109483, PI178884, and PI214049	Colle et al., 2014
Downy mildew	*Cucumis sativu*s L.	PI 197088	Berg, 2020
Cucurbit powdery mildew	*Momordica charantia* L.	THMC 153 and THMC 167	Dhillon et al., 2018
Powdery mildew	*Citrullus lanatus*	PI 632755, PI 386015, PI 189225, PI 346082, PI 525082, PI 432337, PI 386024, and PI 269365	Tetteh et al., 2010
Powdery mildew	*Cucumis sativus* L.	PI 418962, 418964, 432860, 432870, 197085, 197088, 605930, 279465, 288238, 390258, 390266, 330628, 426169, 426170, 321006, 321009, and 321011	Block and Reitsma, 2005
Powdery mildew	*M. charantia var. muricata*	IC 213312, AC-16/1, AC-16/4, AC-16/9, and AC-16/21	Asna, 2018
Powdery mildew	*Cucumis sativu*s L.	PI 197088	Wang Y. et al., 2018
Anthracnose	*Cucumis sativu*s L.	Dual, Regal, Slice, and Gy 3	Wehner and Amand, 1995

#### (b) Breeding for Abiotic Stress Tolerance

Genomic/genetic resources and plant transformation protocols have recently been developed and standardized for cucurbits (Nanasato et al., 2013; Sun et al., 2017; Montero-Pau et al., 2018, 2017). The characterization of the wild relatives of cucurbits have aided in finding their potential use in breeding and other related crop improvement initiatives (e.g., Holdsworth et al., 2016). It was also interesting to note that many wild relatives have multiple stress tolerance capacities, both biotic and abiotic. Potential and documented use of wild cucurbits in breeding with special emphasis on possession of traits such as abiotic and biotic stress tolerance is tabulated in Table 9. It has also been reported that many of the wild cucurbits are under the threat of being endangered, demanding conservation interventions owing to their tolerance potentials (Khoury et al., 2020).

**TABLE 9:** Wild relatives of genus *Cucurbita* and their documented tolerance/resistance potentials.

Taxon	Tolerance/Resistance potentials
*Cucurbita argyrosperma* C. Huber subsp. *sororia* (L. H. Bailey) L. Merrick and D. M. Bates	Resistant to BYMV and TmRSV
*C. cordata* S. Watson	Drought-tolerant; resistant CMV, TRSV, BYMV
*C. digitata* A. Gray	Drought-tolerant; resistant to CMV, TmRSV
*C. ecuadorensis* H. C. Cutler and Whitaker	Resistant to papaya ringspot virus, WMV, powdery mildew, downy mildew
*C. lundelliana* L. H. Bailey	Resistant to SqLCV, CMV, powdery mildew
*C. okeechobeensis* (Small) L. H. Bailey subsp. *Martinezii* (L. H. Bailey) T. C. Andres and Nabhan ex T. W. Walters and D. S. Decker	Resistant to CMV, BYMV, TRSV, bacterial leaf spot, powdery mildew, downy mildew
*C. okeechobeensis* (Small) L. H. Bailey subsp. *okeechobeensis*	Resistant to CMV, BYMV, TRSV, bacterial leaf spot, powdery mildew, downy mildew
*C. palmata* S. Watson	Drought-tolerant; resistant to CMV, TRSV, BYMV, TmRSV
*C. pedatifolia* L. H. Bailey	Drought-tolerant; disease resistance unstudied; potential as bridge species between xerophytic and mesophytic species
*C. radicans* Naudin	Drought-tolerant; resistant to CMV, TmRSV; BYMV
*C. x scabridifolia* L. H. Bailey	Drought-tolerant

Concerted efforts have been made to identify and characterize the potential species and genotypes, among the different cucurbits, considered tolerant to drought or heat or a combination of both stresses (Saroj and Choudhary, 2020; Mkhize et al., 2021). The potential tolerant sources have been tabulated in Table 10.

**TABLE 10:** Abiotic stress tolerant genotypes/species of cucurbits.

Abiotic stress	Crop	Resistant genotype identified	Source
Drought	*Cucumis melo* var. *momordica*	VRSM-58	Bihar, India
	*Cucumis melo* var. *chate*	Arya	Rajasthan and Haryana, India
	Watermelon	AHW-65; Thar Manak	ICAR-CIAH, Bikaner, India
Heat	*Cucumis melo* var. *callosus*	AHK-119, AHK-200	ICAR-CIAH, Bikaner, India
	*Lagenaria siceraria*	Thar Samridhi	
	*Luffa acutangula*	Thar Kami	
	*Luffa cylindrica*	Thar Tapish	
	*Cucumis melo* var. *utilissimus*	Thar Sheetal, AHC-2, AHC-13	
	*Citrullus lanatus*	AHW-19, AHW-66, Thar Manak	
	*Cucumis melo*	Mln 28, CU 311	Turkey
	*Cucumis melo* var. *flexuosus*	Armenian Cucumber	Egypt
Drought and Heat	*Cucumis melo* var. *callosus*	AHK-119, AHK-200	ICAR-CIAH, Bikaner, India
	*Cucumis melo* var. *momordica*	AHS-10, AHS-82	
Drought and Salt	*Cucumis melo* Cv. *reticulatus*	Galia type Cv.1	Pre commercial melons from Enza Zaden, Netherlands
	*Cucumis melo* Cv. *inodorus*	Piel de Sapo Cv. 3	
Heavy metal tolerance (Pb)	*Citrullus lanatus*	NBT, ZM5	–

**FIGURE 1:**
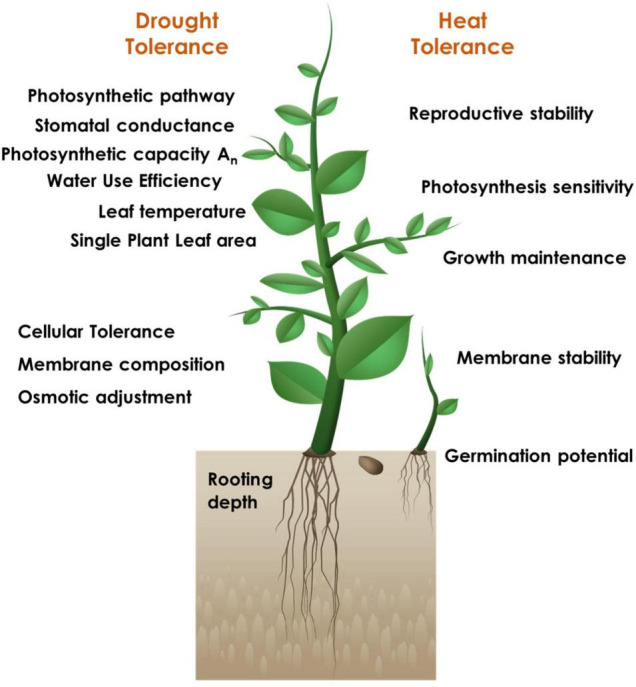
Physiological trait-based screening for identification of drought and heat stress tolerant sources.

The successful strategy for identifying the candidate tolerant sources and developing elite donors is inclined towards a physiogenetic approach including careful analysis of the key physiological traits distinctly critical for each stress (Figure 1) and subsequent characterization of the genetic basis for the respective trait manifestation. However, it is pertinent that there may be common physiological traits that can be capable enough to confer tolerance to multiple stresses. Abiotic stress tolerance, in particular, is a complex trait; the component primary, secondary (constitutive or induced) and integrative traits will have their distinct individual relevance under different stresses, along with their contributory significance.

#### (c) Molecular Breeding and Biotechnological Approaches for Crop Improvement in Cucurbits

Strategies adopted for development of stress tolerant genotypes in cucurbits including conventional breeding, propagation techniques, mitigation strategies etc. have contributed immensely to the successful cultivation of members of the cucurbit family. However, traditional approaches are often time consuming and restricted by the available variation in the gene pool. Biotechnological interventions can result in rapid and sustainable development of crop varieties having high quality and stress tolerance.

##### (i) Genome Sequencing, Mapping and Marker Assisted Selection

Genome sequencing facilitates all subsequent analyses of genome structure, organization and function. Genome sequences have been published for major cucurbit family members like cucumber (Huang et al., 2009; Osipowski et al., 2020), melon (Garcia-Mas et al., 2012; Ruggieri et al., 2018), water melon (Guo et al., 2013), zucchini (Montero-Pau et al., 2018; Xanthopoulou et al., 2019), *C. maxima* (Sun et al., 2017), *C. moschata* (Sun et al., 2017), bottle gourd (Wu et al., 2017), wax gourd (Xie et al., 2019) etc. Genomic information has facilitated the discovery of genes and pathways associated with several stress response pathways leading to the development of stress tolerant varieties or genotypes.

In cucumber, several quantitative trait loci (QTLs) associated with resistance to virus, fungi and bacteria have been mapped. QTLs associated with abiotic stress tolerance like cold, water stress, temperature (Dong et al., 2020; Liu et al., 2021c), drought, salt (Liu et al., 2021a) etc. have also been identified in cucumber which can be utilized in breeding programs. Phytophthora crown rot resistance in *C. moschata*. was detected on chromosome 4 (QtlPC-C04), 11 (QtlPC-C11), and 14 (QtlPC-C14) by bulk segregant analysis and potential linked markers for utilization in marker assisted selection (MAS) (Ramos et al., 2020). A genome-wide association study (GWAS) based on 5,330 single-nucleotide polymorphisms (SNPs) in bottle gourd accessions detected *HG_GLEAN_10011803* to be likely the major-effect candidate gene for resistance against FW in bottle gourd (Yanwei et al., 2021). Wang Y. et al. (2018) identified three major-effect contributing QTLs for downy mildew resistance viz., *dm5.1*, *dm5.2*, and *dm5.3* and a major-effect QTL *pm5.1* for powdery mildew resistance in cucumber. CsGy5G015660, encoding a putative leucine-rich repeat receptor-like serine/threonine-protein kinase (RPK2), was identified as a strong powdery mildew resistance candidate gene in a Korean cucumber inbred line, by genome wide SNP profiling and corresponding RT-PCR analyses (Zhang C. et al., 2021).

SSR marker ECM230 linked to the major QTL in melon (*Cucumis melo* L.) was reported to be useful in selection for resistance to CCYV (*Cucurbit chlorotic yellows virus*) (Kawazu et al., 2018). Two additive QTLs affected the whitefly attack and a major QTL that reduces acceptance by *Aphis gossypii* and 10 genome locations on five linkage groups involved in resistance to hemipterans in melon have been identified (Boissot et al., 2010). In cucumber, resistance to Watermelon mosaic virus (WMV) is controlled by a single recessive gene designated as wmv02245 and was mapped to chromosome 6 (Chr.6) (Tian et al., 2016). The bottle gourd genome sequence has facilitated the mapping of Prs, conferring Papaya ring-spot virus (PRSV) resistance, on chromosome 1 and the potential of a CAPS marker tightly linked to the Prs locus in marker-assisted selection of PRSV resistance in bottle gourd has been demonstrated (Wu et al., 2017). Zhang et al. (2014) identified SSR17631 marker, which could be used to screen cucumber resources with Fusarium wilt resistance in molecular marker-assisted selection breeding. The identified QTLs and associated markers can be effectively utilized in screening, selection and gene pyramiding for multiple stress tolerance in cucurbits.

##### (ii) Transgenic Development for Crop Improvement

Majority of the transgenics developed in cucurbits are for development of virus resistance (Gaba et al., 2004). Transgenic watermelon carrying a single chimeric transgene comprising a silencer DNA from the partial N gene of Watermelon silver mottle virus (WSMoV) fused to the partial coat protein (CP) gene sequences of CMV, Cucumber green mottle mosaic virus (CGMMV) and WMV demonstrated that fusion of different viral CP gene fragments in transgenic watermelon contributed to multiple virus resistance via RNA-mediated post-transcriptional gene silencing (PTGS) (Lin et al., 2012). Transgenic cucumber and melon lines harboring a hairpin construct of the Zucchini yellow mosaic potyvirus (ZYMV) HC-Pro gene displayed resistance to systemic ZYMV infection (Leibman et al., 2011). Transgenic oriental melon carrying untranslatable chimeric DNA with partial CP sequences of ZYMV and PRSV caused RNA-mediated PTGS conferring high degrees of resistance to ZYMV and PRSV W in *C. melo* (Wu et al., 2010). Transgenic watermelon with resistance to CMV infection was developed by expressing artificial microRNAs that target CMV 2a/2b genes (Liu et al., 2016).

##### (iii) Non-transgenic Biotechnological Approaches

Heavy restrictions placed on genetically modified organisms (GMOs) have resulted in the adoption of non-transgenic approaches in crop plants. CRISPR/Cas9, the novel and efficient tool for genome editing, was used in cucumber for the disruption of the *eIF4E* for the development of virus-resistant plants without otherwise affecting the plant genome (Chandrasekaran et al., 2016). Use of CRISPR/Cas9-mediated gene modification for *Clpsk1* loss-of-function in watermelon seedlings made them more resistant to infection by *Fusarium oxysporum* f. sp. *niveum* indicating its effectiveness for watermelon improvement (Zhang et al., 2020). Strategies using the ability of dsRNAs to activate the plant RNA silencing mechanism has also been exploited in cucurbits. Exogenous application of *in vitro*-produced dsRNA molecules derived from the HC-Pro and CP genes of ZYMV, conferred significant protection ZYMV in watermelon and cucumber (Kaldis et al., 2018).

### C. Novel Stress Tolerance Pathways and Mechanisms- the “Omics” Way

The evolution of high throughput next generation sequencing technologies have aided in the generation of immense omics resources for unraveling the more complex stress acclimation responses in cucurbits as has been demonstrated in many other crop species in the past two decades. The Cucurbit Genome Database (CuGenDB) developed by the Fei Lab at Boyce Thompson Institute, United States, serves as the integral portal for functional and comparative genomics (Zheng et al., 2019). The team has added on more tools to their armory with the development of CucCAP (Grumet et al., 2020), which helps in harnessing genomic resources for disease resistance and management in cucurbit crops. The expression repertoire in terms of transcriptomic and proteomic studies have also found place in the cucurbit quest for tolerance to abiotic and biotic stresses. RNA sequencing attempts have been made to prospect genes involved in long-term waterlogging tolerance in cucumber, unraveling transcript abundance specified to “plant hormone signal transduction pathway” in the “environmental information processing” category (Kreska et al., 2021). Salt stress specific transcriptomic analysis revealed the differential regulation of genes associated with carbon metabolism, biosynthesis of amino acids, carbon fixation in photosynthesis, nitrogen metabolism and fatty acid degradation in cucumber (Jiang et al., 2020). This study assumes significance in the context of the role of H_2_S in alleviating salinity stress wherein, proteome analysis indicated differential regulation of proteins involved in sulfur metabolism such as Cysteine synthase 1, Glutathione S-transferase U25-like, Protein disulfide-isomerase, and Peroxidase 2 (Jiang et al., 2020). WRKY transcription factors were reported to regulate downy mildew resistance in cucumber as evidenced by the higher expression of pattern recognition receptor (PRR) proteins unravelled by transcriptome analysis (Gao et al., 2021). Phenylpropanoid biosynthesis pathway emerged as a key regulator of resistance to *Corynespora cassiicola* stress in cucumber as revealed by transcriptome and miRNA analysis (Wang X. et al., 2018). Sucrose biosynthesis and ABA signal transduction were reported to be the key molecular regulations under drought stress, specifically induced after 4 days of drought stress in cucumber (Wang M. et al., 2018). Organellar genome influence, with special emphasis to chloroplastic and mitochondrial genomes in regulation of multiple traits have been highlighted by interventions brought about by next generation sequencing and *omics* in cucumber (*Cucumis sativus* L.) (Pawełkowicz et al., 2016).

In *Cucurbita pepo* subsp. *pepo*, down regulation of SA precursor related enzyme, *CpPAL* (Phenyl ammonia lyase) was found to be associated with susceptibility, while defensin overexpression was found to be related to tolerance (Ayala-Doñas et al., 2021). Targeted metabolomics studies have been attempted to understand the response of cucumber to silver and silver nanoparticles (Zhang et al., 2018), sulfur (Liu et al., 2021b) and elevated atmospheric CO_2_ (Li et al., 2018). An interdisciplinary approach involving different fields of plant sciences to culminate in adopting ionomics as an integrated assessment of elemental accumulation, will hold potential because molybdenum and iron are reported to mutually govern their homeostasis in cucumber (*Cucumis sativus*) plants (Vigani et al., 2017; Pita-Barbosa et al., 2019).

### D. Stress Mitigation Strategies for Cucurbits by Exogenous Amelioration

Cucurbits are a class of vegetables often grown in hot and dry tropics, making them vulnerable to the exposure of multiple stresses. The knowledge and information on the different stress adaptive traits and mechanisms have paved the way for employment of different biostimulants and chemical ameliorants for sustainable management of different stressors. Mycorrhizal associations in cucurbits have been proven to be beneficial both under optimal and stressful conditions. The symbiotic interaction with arbuscular mycorrhizal fungi (AMF) has profound influence when multiple stresses occur at the same time. There are commercial examples of mycorrhizal consortia such as MycoApply ^®^, a four-species consortium, which facilitates nutrient and water uptake^1^. A consortium of three plant growth-promoting rhizobacterium (PGPR) strains (*Bacillus cereus* AR156, *Bacillus subtilis* SM21, and *Serratia* sp. XY21), has been reported to confer systemic tolerance to drought stress in cucumber, by maintaining assimilation and growth vigor and offering protection against oxidative stress damage (Wang C. J. et al., 2012). Humic acid is a highly beneficial biostimulant used in different crops to stimulate shoot and root growth and enhance tolerance to stresses, which has also been demonstrated in cucumber to influence yield and mineral nutrient uptake under salinity stress exposure (Demir et al., 1999). Foliar spray of Moringa leaf extract was found to be beneficial in enhancing growth, harvest index, WUE, photosynthetic stability, osmoregulation and membrane stability in *Cucurbita pepo* under drought stress (Abd El-Mageed et al., 2017). Similarly, under salinity stress, seed treatment/irrigation with a bacterial consortia of *Bacillus species, Bacillus pumilis, Trichoderma harzianum, Paenibacillus azotoformans*, and *Polymyxa* plays a role in maintaining growth by regulating ion homeostasis in *Cucurbita pepo* (Yildirim et al., 2006). Soil amelioration with *Ascophyllum nodosum* was beneficial in *Cucumis sativus* against salinity stress, which helped in maintaining fruit yield (Demir et al., 1999). Resistance against *Fusarium oxysporum* induced wilt in cucumber was effectively enhanced by a combination treatment with GAWDA ^®^ (an antioxidant formulation designed and patented in Egypt) and an AMF consortia (Elwakil et al., 2013), and exogenous nitrate nutrition operating through modulation of photorespiration (Sun et al., 2021).

Brassinosteroids (BR) are naturally occurring plant steroids with growth regulatory potential, which has been reported to impart chilling stress tolerance in cucumber (*Cucumis sativus*) by a chemico-genetic regulation of oxidative stress management. BR-induced activation of plasma membrane-bound NADPH oxidase (RBOH) results in the upregulation of signaling molecules in the form of H_2_O_2_, which has a role in activating subsequent stress response pathways (Xia et al., 2009). Exogenous application of 24-Epibrassinolide was found to alleviate the detrimental effects of root zone temperature fluctuations in cucumber seedlings by regulating hormonal and ion homeostasis (Anwar et al., 2018, 2019a,b; Anwar and Kim, 2020). Amelioration of chilling stress in cucumber seedlings by triadimefon (Feng et al., 2003), selenium (Hawrylak-Nowak et al., 2010), and SA (Kang and Saltveit, 2002) have also been reported. Drought tolerance was found to be enhanced in cucumber plants treated with natural carbon materials like shungite, which led to an increase in antioxidant potential thereby reducing cellular damage (Kim et al., 2019). Although there are many strategies employed to combat stress incidence in cucurbits, development of novel technologies in the form of ameliorative treatments can be effective, provided the right traits and target mechanisms come under the purview of the mitigation strategy.

## Future Perspectives in Crop Improvement for Multiple Stress Tolerance in Cucurbits

**FIGURE 2:**
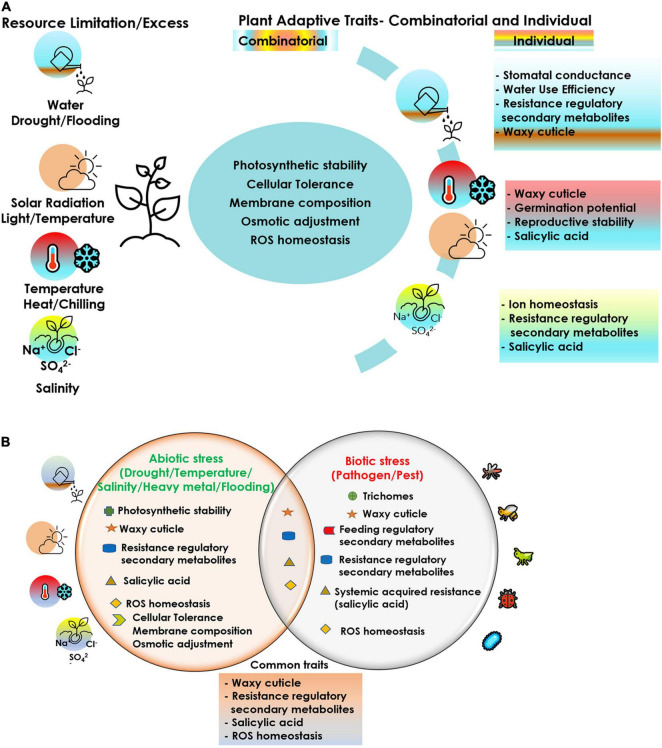
Plant adaptive traits/mechanisms critical under each stressor and prospective trait-stress combinations under co-occurrence of different stresses in cucurbits; **(A)** abiotic–abiotic, **(B)** abiotic–biotic.

Cucurbits are vulnerable to simultaneous exposure of multiple stressors and hence interventions at various levels are imperative to achieve sustained crop production, even under adverse climatic conditions. The choice of the apt component trait for achieving tolerance to a stress episode is the key towards effective crop improvement. Crop improvement relies on the available diversity or creation of diversity as the source of desirable traits, including abiotic and biotic stress resistance. Genetic diversity is facing serious threat due to habitat loss owing to human intervention and climate change. Efforts for collection and conservation of cucurbit germplasm including related species, distant species, wild relatives and landraces needs to be expedited for their utilization in breeding programs, biotechnological approaches and propagation methods. Grafting has evolved as a relatively cheap and quick option for managing the biotic and abiotic stressors. Although a good number of commercial rootstocks have been identified, particularly in the temperate regions, these attempts are very rare in the tropical regions. Hence, systematic testing of genotypes and wild relatives of cucurbits for their potential use as rootstocks against multiple stresses should be a research priority. Rootstock breeding in cucurbits leading to development of vigorous intra and interspecific hybrid rootstocks conferring tolerance to multiple stresses needs urgent attention. Hence, it is very crucial to screen for the right plant traits/characters/mechanisms for employing any of the prospective strategies discussed in this review. A comprehensive account of the different plant adaptive traits/mechanisms critical under each stressor (Figure 2) can aid in the adoption of the correct trait(s) combination and the approach in the event of a multiple stress exposure. With climate change posing novel, varied and often multiple stresses to the crops, strategies for screening for multiple stress resistance needs to be evolved and employed. Even a moderate level of resistance for multiple stress factors can confer tolerance to stresses through cross-talk between various pathways and mitigate their damaging effect. Targeted manipulation of tolerance traits will be possible if a better understanding of the combined stress effects is realized. Breeding approaches should focus on gene pyramiding for multiple stress tolerance in cucurbits through a combination of conventional and biotechnological approaches. Robust markers, genomic prediction tools and phenotyping facilities need to be developed for rapid screening and identification of stress tolerant genotypes in cucurbits. Previous attempts in this direction in vegetable crops like onion, including gene prospecting for drought tolerance (Kutty et al., 2012) and molecular marker identification by bulk segregant analysis of drought tolerance in F2 population (Kutty et al., 2013), concerted with physiological, morphological and biochemical characterization of drought stress response in different onion cultivars (Kutty et al., 2014) have been promising. Genes/gene products regulating multiple molecular mechanisms thus identified can be combined together by novel gene stacking approaches to alter multiple traits required for combined stress tolerance, as has been demonstrated in model crop plants (Vemanna et al., 2013; Pruthvi et al., 2014; Parvathi et al., 2015). Multiple trait manipulation employing a ‘super regulatory gene/QTL’ capable of coordinating numerous trait-related genes can help in developing genotypes tolerant to multiple stresses (Parvathi and Nataraja, 2017; Parvathi et al., 2021). Such initiatives would help in congregating the indispensable traits in an ideal genotype/accession, that would be critical for crop acclimation under a combination of stress challenges. Advanced gene editing technologies can be of great value in the view of multigenic control over tolerance in a multiple stress scenario. A careful and concerted trait-specific approach for each stress combination is imperative to achieve optimal realizable crop performance.

## Author Contributions

All authors listed have made an equal, direct, and intellectual contribution to the work, and approved it for publication.

## Conflict of Interest

The authors declare that the research was conducted in the absence of any commercial or financial relationships that could be construed as a potential conflict of interest.

## Publisher’s Note

All claims expressed in this article are solely those of the authors and do not necessarily represent those of their affiliated organizations, or those of the publisher, the editors and the reviewers. Any product that may be evaluated in this article, or claim that may be made by its manufacturer, is not guaranteed or endorsed by the publisher.

## Footnotes

1
https://www.valentbiosciences.com/soilhealth/solutions/abiotic-stress-mitigation-for-cucurbit-vegetables/
